# Immunoprognostic model of lung adenocarcinoma and screening of sensitive drugs

**DOI:** 10.1038/s41598-022-11052-8

**Published:** 2022-05-03

**Authors:** Pengchen Liang, Jin Li, Jianguo Chen, Junyan Lu, Zezhou Hao, Junfeng Shi, Qing Chang, Zeng Zeng

**Affiliations:** 1 School of Microelectronics, Shanghai University, Shanghai, 201800 China; 2 Department of Geriatrics, Ren Ji Hospital, Shanghai Jiao Tong University School of Medicine, Shanghai, 200001 China; 3 Institute for Infocomm Research, Agency for Science, Technology and Research, Singapore, 138632 Singapore; 4 Clinical Medicine, Shanghai University of Medicine and Health Science, Shanghai, 201318 China; 5 School of Health Sciences and Engineering, University of Shanghai for Science and Technology, Shanghai, 200093 China; 6 Shanghai Engineering Research Center of Advanced Dental Technology and Materials, Shanghai Key Laboratory of Stomatology, Shanghai Ninth People’s Hospital, College of Stomatology, Shanghai Jiao Tong University School of Medicine, Shanghai, 200011 China; 7 Clinical Research Center, Jiading District Central Hospital Affiliated Shanghai University of Medicine & Health Sciences, Shanghai, 201800 China

**Keywords:** Data mining, Predictive medicine, Virtual drug screening, Cancer models, Tumour biomarkers

## Abstract

Screening of mRNAs and lncRNAs associated with prognosis and immunity of lung adenocarcinoma (LUAD) and used to construct a prognostic risk scoring model (PRS-model) for LUAD. To analyze the differences in tumor immune microenvironment between distinct risk groups of LUAD based on the model classification. The CMap database was also used to screen potential therapeutic compounds for LUAD based on the differential genes between distinct risk groups. he data from the Cancer Genome Atlas (TCGA) database. We divided the transcriptome data into a mRNA subset and a lncRNA subset, and use multiple methods to extract mRNAs and lncRNAs associated with immunity and prognosis. We further integrated the mRNA and lncRNA subsets and the corresponding clinical information, randomly divided them into training and test set according to the ratio of 5:5. Then, we performed the Cox risk proportional analysis and cross-validation on the training set to construct a LUAD risk scoring model. Based on the risk scoring model, patients were divided into distinct risk group. Moreover, we evaluate the prognostic performance of the model from the aspects of Area Under Curve (AUC) analysis, survival difference analysis, and independent prognostic analysis. We analyzed the differences in the expression of immune cells between the distinct risk groups, and also discuss the connection between immune cells and patient survival. Finally, we screened the potential therapeutic compounds of LUAD in the Connectivity Map (CMap) database based on differential gene expression profiles, and verified the compound activity by cytostatic assays. We extracted 26 mRNAs and 74 lncRNAs related to prognosis and immunity by using different screening methods. Two mRNAs (i.e., KLRC3 and RAET1E) and two lncRNAs (i.e., AL590226.1 and LINC00941) and their risk coefficients were finally used to construct the PRS-model. The risk score positions of the training and test set were 1.01056590 and 1.00925190, respectively. The expression of mRNAs involved in model construction differed significantly between the distinct risk population. The one-year ROC areas on the training and test sets were 0.735 and 0.681. There was a significant difference in the survival rate of the two groups of patients. The PRS-model had independent predictive capabilities in both training and test sets. Among them, in the group with low expression of M1 macrophages and resting NK cells, LUAD patients survived longer. In contrast, the monocyte expression up-regulated group survived longer. In the CMap drug screening, three LUAD therapeutic compounds, such as resveratrol, methotrexate, and phenoxybenzamine, scored the highest. In addition, these compounds had significant inhibitory effects on the LUAD A549 cell lines. The LUAD risk score model constructed using the expression of KLRC3, RAET1E, AL590226.1, LINC00941 and their risk coefficients had a good independent prognostic power. The optimal LUAD therapeutic compounds screened in the CMap database: resveratrol, methotrexate and phenoxybenzamine, all showed significant inhibitory effects on LUAD A549 cell lines.

## Introduction

In China, lung cancer has the highest incidence and mortality among male malignancies ,and the second highest incidence among female malignancies, second only to breast cancer^1^.

There are two subtypes of lung cancer, the first is small cell lung cancer and the second is non-small cell lung cancer (NSCLC), of which NSCLC includes two main types of lung adenocarcinoma (LUAD) and squamous carcinoma^2^. LUAD in lung cancer accounts for 40% of all histological types^3^. The incidence of LUAD is increasing year by year, with a trend toward younger age, rapid onset, few initial symptoms, high mortality, and poor prognosis^4^.

Advances in molecularly targeted therapies have led to a major shift in the LUAD treatment paradigm, with LUAD treatments demonstrating higher positive response rates compared to conventional cytotoxic chemotherapy^5^. Through the genetic testing of LUAD patients, the most common lung cancer driver genes are identified as EGFR, ALK, ROS1, and BRAF^6^. Recent studies show that increased expression of PITX2 and CENPH genes is positively correlated with the development of LUAD^7^. In addition, some specific long-chain non-coding RNAs (lncRNA) show high specificity in prostate cancer, breast cancer, and lung cancer^8^. These studies found that lncRNA CASC9.5 regulates the expression of cyclin D1, E-cadherin, N-cadherin, and $\beta$-catenin protein. CASC9.5 induces cells to stay in the G1 phase and promotes the proliferation and metabolism of LUAD cells^9^. Although targeted therapies have been successful in the treatment of LUAD, resistance to EGFR targeted therapies (such as gefitinib) is still a problem^10,11^. Moreover, a large proportion of LUAD still lacks targeted therapy^12^. Therefore, exploring new biomarkers and prognostic factors has become a research trend in the era of precision medicine.

In recent years, through the analysis of the public database, more prognostic biomarkers of LUAD have been identified^13–15^. In^16^, Liu et al. analyzed the expression profiles of LUAD patients in the database and recognized glycolytic genes (i.e., AGRN, AKR1A1, DDIT4, and HMMR) that are closely associated to the prognosis of LUAD patients. In^17^, Jiang et al. conducted multiple differential gene analyses related to LUAD and then developed a risk model composed of 10 genes. The above studies have proved the great potential of using the open database to develop PRS-model. Therefore, it is inevitable to continue to investigate the polygenic characteristics related to the prognosis of LUAD.

In this work, we collected the transcriptome and clinical data with LUAD from the TCGA database^18^. The TCGA database contains information about various human tumors, including molecular changes at the DNA, RNA, protein, and epigenetic levels^19^. We used mRNAs and lncRNAs related to prognostic immunity to construct a LUAD risk scoring model, and used it to predict and evaluate the survival rate of patients with LUAD. CMap^20^ is a drug gene expression database, which provides an possibility to comprehend the mechanism of action of unknown compounds and find new indications for existing drugs^21^. In addition, We submitted the differential gene expression profiles in distinct risk patients with LUAD to the CMap database and matched the drugs with opposite or similar gene expression profiles. Drugs with opposite gene expression profiles are considered as potential treatments for the disease^22^.

The purpose of this study was to establish a prognostic model of LUAD , to screen possible drugs for the treatment of LUAD in the CMap database , which using the differential gene expression profiles of distinct risk groups distinguished by the model.

## Results

### Prognosis-related Immune MRNAs and LncRNAs screening

The LUAD transcriptome data was converted by gene name and divided into mRNAs and lncRNAs data. Using the IMMPORT database^23^, we further extracted 1354 immune-related mRNA data from the LUAD mRNA data. Using the Weighted Correlation Network Analysis (WGCNA) method to analyze the expression profile data containing 1354 immune-related mRNAs and the corresponding clinical information of the samples. In this way, a total of 387 mRNAs associated to the patient’s survival status and survival time were extracted. We further performed univariate COX regression(UCR) analysis on the expression profile data containing 387 mRNAs and their corresponding patient survival status and survival time. Then, we screened a total of 26 immune mRNAs associated to prognosis, namely: HLA-DOB, KLRC2, KLRC3, RFX5, RFXAP, RAET1E PTGDS, GDF15, F2RL1, CD40LG, ARRB1, CD19, CD22, PTPN6, PRKCB, XCR1, IL16, IL24, HTR3A, IL11RA, IL1R2, SORT1, ITGAL, MAP3K8, JAG1, and OXTR.

We also used the UCR analysis method to compare the lncRNAs expression profile data of LUAD with the prognostic-related immune mRNAs expression data screened. After that, we performed the correlation coefficient test to screen out 207 lncRNAs related to the above-mentioned mRNAs. Through the UCR analysis on the expression data of the 207 lncRNAs, the survival time, and survival status of the corresponding patients, we could screen a total of 74 lncRNAs that were significantly related to the prognosis. Therefore, by using different statistical methods, we finally screened a total of 26 mRNAs and 74 lncRNAs related to prognosis and immunity.

### Prognostic modeling of LUAD based on immune-related mRNAs and LncRNAs

We used the training set to construct the lasso model, in which the Cox risk proportional model was selected to fit the data, and the minimum point of the model error was 4 through cross-validation. Two mRNAs (i.e., KLRC3 and RAET1E) and two lncRNAs (i.e., AL590226.1 and LINC00941) were further screened to construct PRS-model. After the above calculation, we could formulate the PRS-model as:

1 $$\begin{aligned} \begin{aligned} Risk score =\,&[AL590226.1] \times (-0.26449606) + [LINC00941] \times (0.244434874) \\&+ [KLRC3] \times 0.11099252 + [RAET1E] \times (0.19868693) \end{aligned} \end{aligned}$$

**Figure 1:**
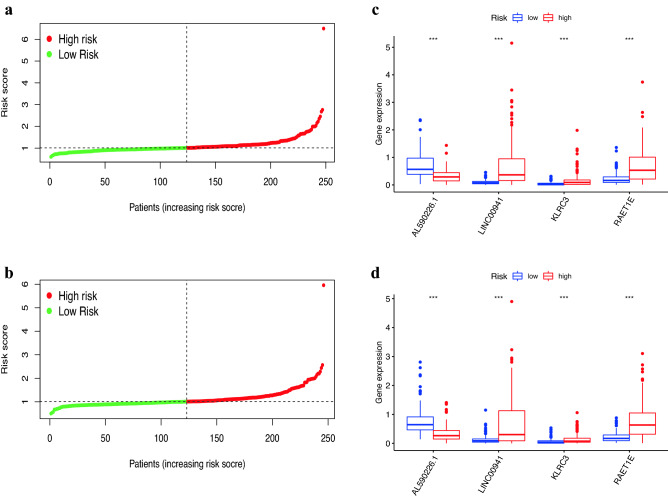
Model-based differentiation of distinct risk populations. (**a**) Distinct risk group division in the training set. (**b**) Distinct risk group division in the test set. (**c**) The expression of modeled mRNAs and lncRNAS in the two groups in the train set. (**d**) The expression of modeled mRNAs and lncRNAS in the two groups in the test set.

According to the PRS-model formula, the median risk value of the training set was 1.01056590, and the median value of the test set was 1.00925190. Base on this, the patients in the training and test sets were divided into distinct risk groups, respectively. The classification results of the distinct risk groups are shown in Fig. 1a, b. The expression of the KLRC3 and RAET1E mRNAs and the AL590226.1 and LINC00941 lncRNAs as the Fig. 1c, d shows. These mRNAs and lncRNAs were involved in the building of the model, and there were marked differences among the distinct risk groups.

### Evaluation of model reliability based on clinical information

**Figure 2:**
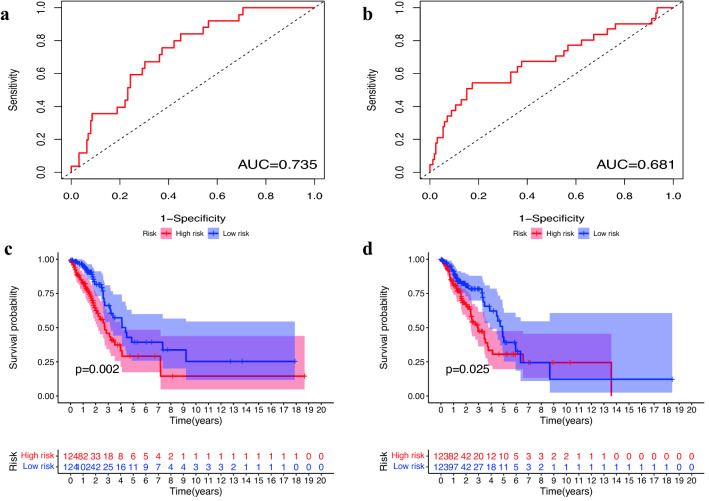
Evaluation of prognostic ability of the model based on clinical information. (**a**) One-year ROC curve in the training set. (**b**) One-year ROC curves in the test set. (**c**) Analysis of survival differences between the distinct risk populations in the training set. (**d**) Survival difference analysis between the distinct risk groups in the test set.

We plotted the one-year ROC for the training and the test sets, and calculated the area under the curve. As shown in Fig. 2a, b, the ROC area was 0.735 for training , and 0.681 for the test. Therefore, the model had good performance in predicting the one-year survival rate of patients.

Survival analysis results are in the Fig. 2c, d. The PRS-model distinguished between different risk groups of patients in the training, and the survival rate was significantly different (*p* = 0.020). In addition, patients in the low-risk group had a better survival rate. The results were consistent in the training set. The experimental results indicated that PRS-model had better performance in predicting the survival rate.

**Figure 3:**
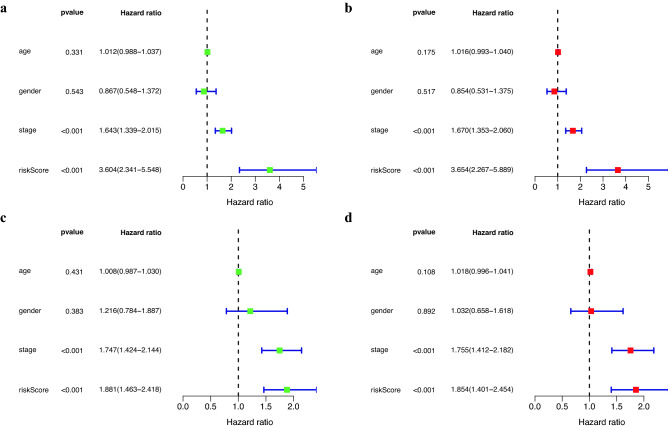
Evaluation of the independent prognostic ability of the risk scoring model. (**a**) Results of univariate COX regression of risk scores with clinical information in the training set. (**b**) Results of multi-factor COX regression of risk scores and clinical information in the training set. (**c**) Results of univariate COX regression in the test set. (**d**) Results of multifactor COX regression in the test set.

As shown in Fig. 3, in the COX regression analysis, the *P*-values of the risk score in both of the training set and the test set were less than 0.001. The above results revealed that the risk score could be used as an separate prognostic factor, and the risk scoring model had a good prognostic ability.

### Differences in immune microenvironment between distinct risk groups

**Figure 4:**
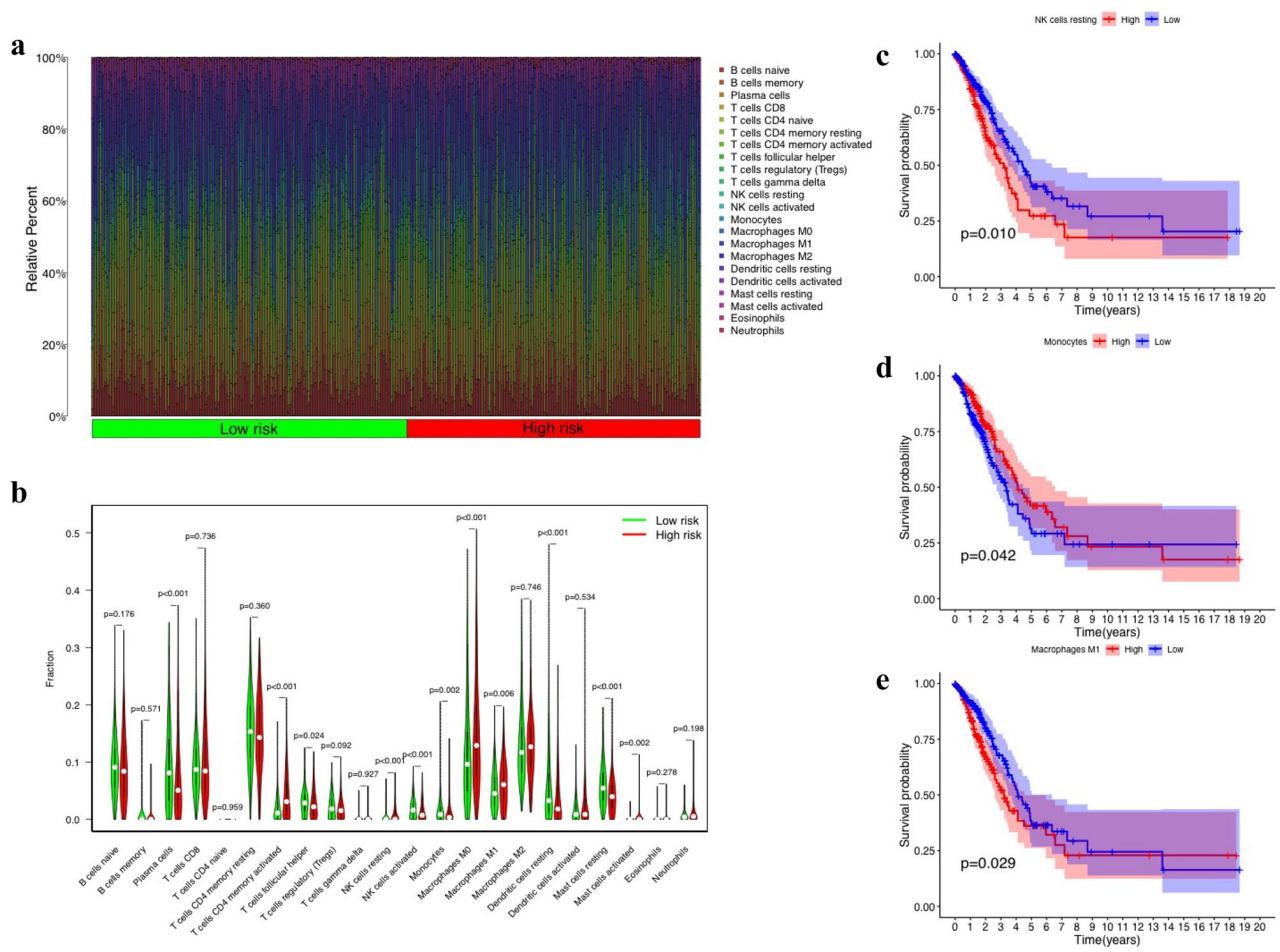
Differences in the immune microenvironment of the high and low-risk patients. (**a**) Heat map of immune cell expression between the high and low-risk populations. (**b**) Statistics of differences in immune cell expression between two groups. (**c**, **d**, **e**) NK cells, monocytes, and Macrophages m1 were analyzed for differences between groups, respectively.

The heat map of 22 immune cell infiltration is shown in Fig. 4a, and the analysis results of variance are shown in Fig. 4(b). The resting NK cell group, activated NK cell group, monocyte group, M0 macrophage group, M1 macrophage group, etc., showed statistically significant differences in expression between high and low risk groups. After co-expression with survival time and filtering according to *P* < 0.05, 3 out of 22 immune cells were found to be associated with prognosis, such as M1 macrophages, resting NK cells, and monocytes. In addition, the survival analysis results showed that patients’ years of survival were all related to three types of immune cells. Specifically, the *P*-value of resting NK cells was 0.01 (as shown in Fig. 4c), the *P*-value of monocytes was 0.042 (as shown in Fig. 4d), and the *P*-value of M1 macrophages was 0.029 (Fig. 4e).

Among them, in the group with lower expression of M1 macrophages and resting NK cells, patients with LUAD had longer survival times, while longer survival times were observed in patients with up-regulated monocyte expression.

### Differentially expressed genes and functional annotation of distinct risk groups

**Figure 5:**
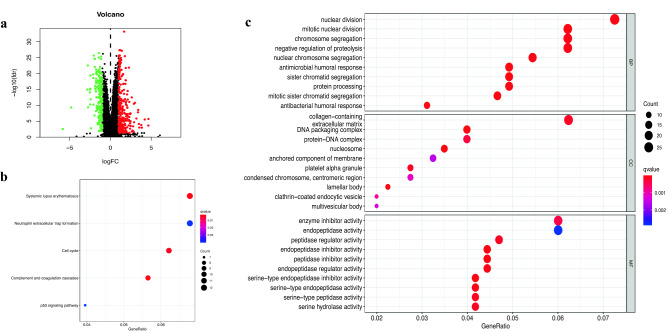
DEGs and enrichment analysis between the distinct risk populations (*a*) Volcano map of DEGs between two groups. (*b*) KEGG analysis of DEGs. (*c*) GO analysis of DEGs.

We performed the differential gene analysis on the distinct risk groups, and detect a total of 290 differentially up-regulated genes and 191 differentially down-regulated genes in the high risk group, (Fig. 5a). We also performed the functional annotation analysis of differential expressed genes(DEGs). The KEGG analysis results showed that the main enrichment pathways were systemic lupus erythematosus, cell cycle, complement, and coagulation cascade, as shown in Fig. 5b. We also performed the GO enrichment analysis by considering the cellular composition (CC), molecular function (MF), and biological process (BP), (Fig. 5c). The GO enrichment analysis results showed that the mRNA differences of CC were mainly concentrated in collagen (including extracellular matrix), DNA packaging complex, and protein-DNA complex. MF mainly manifests as enzyme inhibitor activity. BP mainly includes nuclear division, mitotic nuclear division, chromosome segregation, and negative regulation of proteolysis.

### CMap drug screening and cytological activity validation

Based on the significant difference in mRNA data between the distinct risk group, a total of 1309 drugs were screened in the CMap database^20^. Among them, 48 drugs with *P*-value < 0.05 and enrichment score < 0 were considered as potential drugs to inhibit the expression of high-risk genes. Table 1 lists the results of the top 20 drugs.

**Table 1:** CMap database drug screening.

Name	Rank	Enrichment	*P* value	Specificity
Resveratrol	1	− 0.838	0.000	0.000
Methotrexate	2	− 0.748	0.000	0.000
Phenoxybenzamine	3	− 0.904	0.000	0.009
Thiostrepton	4	− 0.894	0.000	0.009
15-delta prostaglandin J2	5	− 0.491	0.001	0.113
Xylometazoline	6	− 0.848	0.001	0.000
6-bromoindirubin-3’-oxime	7	− 0.667	0.001	0.051
MG-262	8	− 0.892	0.002	0.087
Chlorphenesin	9	− 0.810	0.003	0.010
Bepridil	10	− 0.789	0.004	0.023
Monobenzone	11	− 0.786	0.004	0.014
Irinotecan	12	− 0.861	0.005	0.087
Bupropion	13	− 0.765	0.006	0.006
Megestrol	14	− 0.743	0.009	0.000
Menadione	15	− 0.936	0.009	0.023
Tribenoside	16	− 0.725	0.012	0.000
Chlorcyclizine	17	− 0.603	0.014	0.009
Scriptaid	18	− 0.808	0.014	0.135
Tretinoin	19	− 0.321	0.015	0.184
Luteolin	20	− 0.706	0.016	0.074

**Figure 6:**
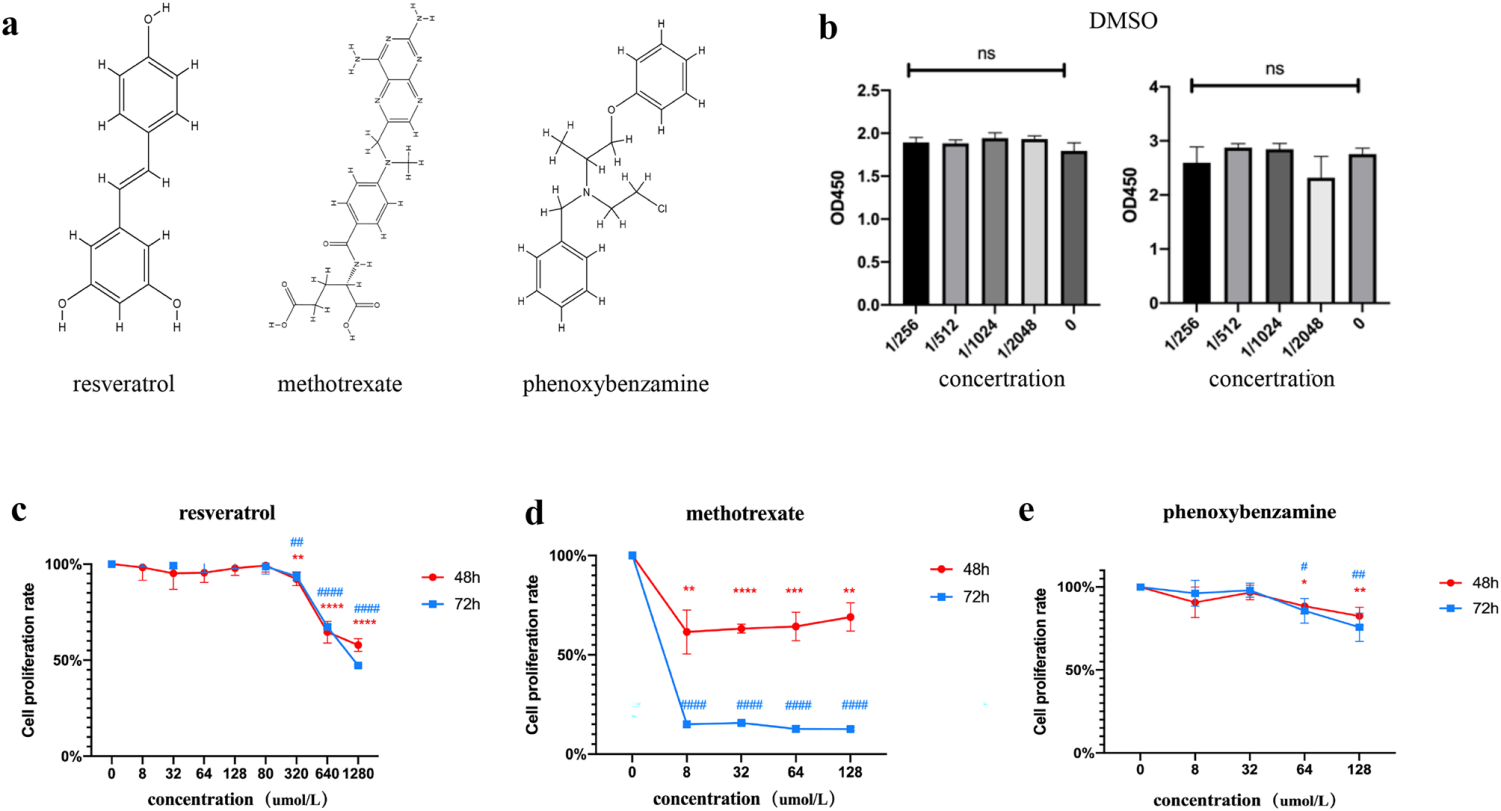
CMap database drug screening and cytological activity validation. (**a**) Optimal compounds and their structures screened from the CMap database. (**b**) DMSO solvent toxicity assay. (**c**) Inhibition rate of the resveratrol compound on A549 cells. (**d**) Inhibition rate of the methotrexate compound on A549 cells. (**e**) Inhibition rate of the phenoxybenzamine compound on A549 cells.

Finally, we screened the top three drugs with the largest differences to verify their inhibitory effects on LUAD cell proliferation. The three drugs were resveratrol, methotrexate, and phenoxybenzamine, and their structures are shown in Fig. 6a.

When A549 cells were treated with distinct concentrations of DMSO for 48 hours and 72 hours, and the DMSO concentration was less than 1/256, cell proliferation was not affected (Fig. 6b). We set the concentration of the original drug solution to 125 mg/mL, that was, when the compound concentration was less than 488 μg/mL, the inhibitory effect of the compound was not affected by DMSO, which could objectively reflect the inhibitory effect of the drugs on A549 cells. The maximum concentration of resveratrol 1280 μmol/L (292 μg/mL), methotrexate 128 μmol/L (58 μg/mL), and phenoxybenzidine 128 μmol/L (43 μg/mL) were all less than 488 μg/mL, so the interference of DMSO was excluded, and the result was the effect of the drug itself.

As shwon in Fig. 6c, resveratrol had no effect when the concentration was lower than 320 μmol/L, and the cell proliferation rate was 67% at 640 μmol/L . In addition, in Fig. 6d, methotrexate had the best inhibitory effect on the proliferation of A549 cells. After 8 μmol/L treatment, the proliferation rate of A549 cells was 61% at 48 hours and only 15% at 72 hours. As shown in Fig. 6e, for phenoxybenzidine, the proliferation rate of A549 cells was 88% at 64 μmol/L and 82% at 128 μmol/L. The results were similar at 48 hours and 72 hours.

## Discussion

In this research, we screened the mRNAs and lncRNAs related to prognosis and immunity through the TCGA and IMMPORT databases^18,23^. Using the COX risk regression model to constructed the PRS-model for LUAD. Through the AUC analysis and independent prognostic analysis, we found that the PRS-model has good prognostic ability.

Survival of cancer patients is an important indicator to respond to cancer progression, and how to predict survival is currently a key concern in the field of bioinformatics. In this study, the established PRS-model distinguished the present high- and low-risk groups with significant survival differences, which suggests that our PRS-model has a good predictive ability for patient survival.

The characteristic factors involved in model construction are KLRC3, RAET1E, AL590226.1, and LINC00941. In^24^, Li et al. performed comprehensive analysis on multi-omics data, and identified KLRC3 as a key node in the regulatory network of immune phenotype-related genes in LUAD. In^25^, Cho et al. found that the expression of RAET1E in cervical cancer is higher than that of RAET1E in low-grade CINs, and the lower survival rate of patients is associated with high RAET1E expression. In^26^, Geng et al. constructed an lncRNA prognostic model related to genetic instability in LUAD. Among the 7 lncRNAs involved in the model, they found that AL590226.1 was a protective factor for patient prognosis. In addition, in^27^, Wang et al. analyzed the lncRNA expression and copy number change data (CNA) of patients with LUAD in the TCGA database. The authors found that the CNA of LINC00941 was negatively correlated with the patient’s longer overall survival. The above findings further proved the better prognostic ability of our model.

We conducted an immune microenvironment analysis on the different risk groups distinguished by the PRS-model. Cells that express significant differences between the different risk group are related to the patient’s prognosis, including the M1 macrophages, resting NK cells, and monocytes. Analysis of paraffin-embedded NSCLC samples and their clinicopathological data revealed that the density of M1 macrophages in tumor vesicles was an independent predictor of survival time for patients with NSCLC^28^. NK cells had the ability to lyse tumor cells without the need to sensitize the host beforehand, and the recognition of target cells by NK cells was strictly regulated by a number of processes that activated and inhibited receptor signal integration^29^.

Based on the differential gene expression profiles of the distinct risk group, the compounds of resveratrol, methotrexate, and phenoxybenzidine score the highest in the CMap drug screening. Moreover, the three compounds showed a significant inhibitory effect on the LUAD A549 cell line.

Resveratrol is a polyphenol stilbene found in rhubarb, grapes,and other substances. It has been found to have an excellent immunomodulatory effect^30^. Resveratrol could up-regulate the expression of caspase-3 and Bax, and down-regulate the expression of Bcl-2. In addition, by increasing the level of p53, the levels of phosphorylated Mdm2 and protein kinase B (Akt) were reduced, leading to apoptosis and autophagy in cancer cells^31^.

Methotrexate inhibited the production of malignant pleural effusion and regulated the tumor microenvironment of patients with advanced lung cancer,CD4+ T cells stimulated by methotrexate release more IL-2, while CD8+ T cells released less IFN-$\gamma$^32^.

Phenoxybenzamine is a non-selective, non-competitive, and irreversible alpha-adrenergic receptor blocker, which can covalently bind to alpha-adrenergic receptors to attenuate catecholamine-mediated vasoconstriction^33^ . It could also inhibit histone deacetylase activity, thus inhibiting the growth of adrenal medullary tumors and pheochromocytoma^34^. However, the efficacy and mechanism of action of these drugs in LUAD were not yet completely clear, and further research was needed.

## Conclusion

In short, we constructed a PRS-model of LUAD based on prognostic mRNAs and lncRNAs, which can independently predict the prognosis. The PRS-model determines the DEGs of the distinct risk groups. In addition, the model was also used to screen potential therapeutic drugs for LUAD. Among them, resveratrol, methotrexate, benbenzamine, and other drugs had inhibitory effects on the proliferation of A549 cell lines.

## Methods

### LUAD clinical and transcriptome data collation

RNA-seq expression profile and Clinical information of LUAD came from the TCGA database. The keywords used in the RNA-seq expression profile data search were “bronchi”, “lung”, “TCGA-LUAD”, “adenoma”, “adenocarcinoma”, “transcriptome analysis”, “gene expression quantification”, and ‘HTSeq-FPKM”. The key words used in the clinical data search were “bronchial”, “lung”, “TCGA-LUAD”, “adenoma”, and “adenocarcinoma, clinical”.

We collected a total of 551 transcriptome data, of which 56 were non-cancer or para-cancer data, and 495 were tumor data. We further collected a total of 522 cases of clinical information corresponding to the transcriptome data, where some clinical information relate to multiple transcriptome data.

### Identification of MRNAs and LncRNAs related to immune and prognostic

**Figure 7:**
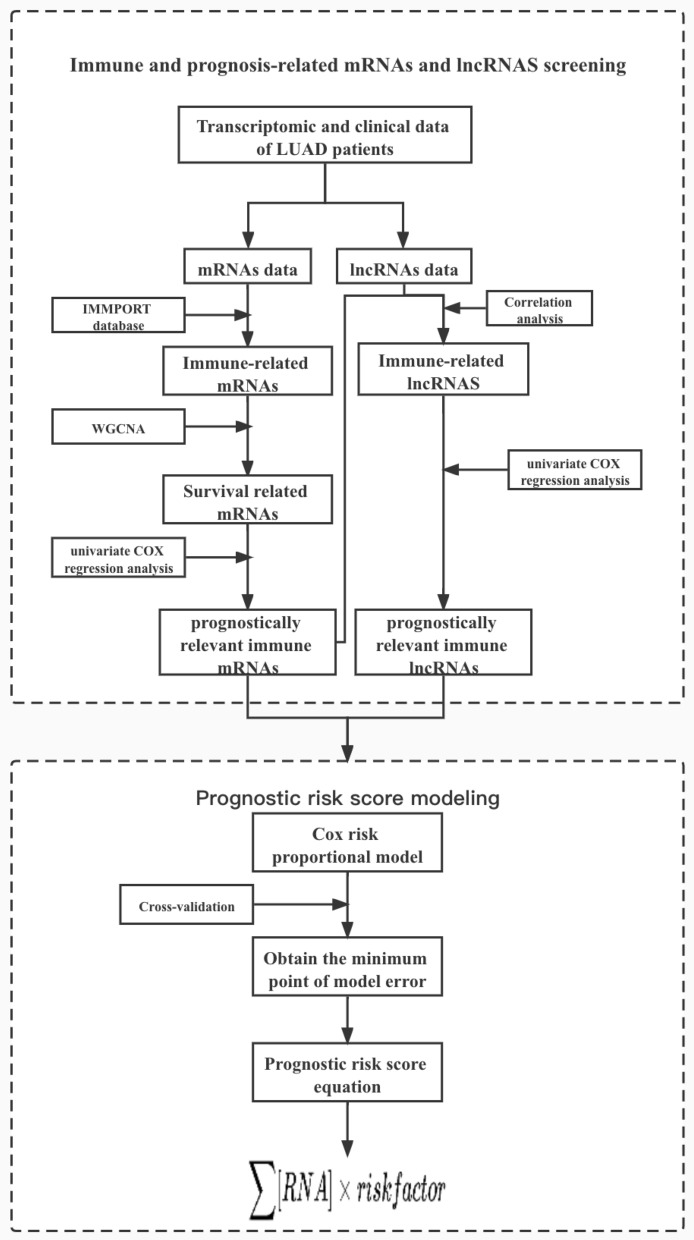
Workflow of immune and prognosis-related mRNAs and lncRNAs screening and prognostic risk scoring model construction. The lung adenocarcinoma data in TCGA database were divided into mRNA sub-dataset and lncRNA sub-dataset, and the mRNAs and lncRNAs associated with immunity and prognosis of lung adenocarcinoma were extracted by various methods such as WGCNA and COX regression, and the lung adenocarcinoma prognostic risk model was further constructed based on these associated factors.

We extracted the data of mRNAs and lncRNAs from the LUAD transcriptome data. The screening process is shown in Fig. 7.

We collected the immune-related gene list from the IMMPORT database^23^, and used the list information to extract the immune-related mRNAs expression profile data from the LUAD mRNAs expression profile data. We performed the WGCNA method and the clustering analysis on immune-related mRNA expression profile data and the corresponding clinical data to extract the survival-related modular genes.

Take *k* as the ergodic, we traversed all genes except *i* and *j* in the gene list, and calculate the correlation coefficient of *k* and *i* and *j* in:

2 $$\begin{aligned} {\text {TOM}}_{i,j}=\frac{\sum _{k \ne i, j}\left( a_{i,k} * a_{k,j}\right) -a_{i,j}}{\min \left\{ \sum _{k \ne i} a_{i,k}, \sum _{k \ne j} a_{k,j}\right\} +1-a_{i,j}}. \end{aligned}$$

If the connection conditions of genes *i* and *j* in the network are similar, the larger $TOM_{i,j}$ is, and it is more likely that genes *i* and *j* are in the same expression module. In the WGCNA analysis, we constructed the optimal scale-free network according to the optimal power value of 3. We set the minimum number of genes for each module to 40, and set the value of Deepsplit to 2, and then drew a dendrogram. we further assemble highly dependent modules, and merge similar modules with a cut height of 0.3 at the boundary. Then, we visualized the correlation with the “Marking Heat Map” function to get the heat map results. We performed UCR analysis on the survival-related gene modules and the survival information to obtain the immune genes related to the prognosis. The parameters of the UCR model were set to *P* < 0.01. We used the UCR analysis method to screen the prognostic-related immune gene expression profile data. By comparing LUAD’s lncRNAs expression profile data with the prognostic-related immune gene expression profile data, We performed correlation test screen the lncRNAs related to the prognostic-related immune genes.

The lncRNAs identified above were subjected to UCR analysis (*P* < 0.05) with We subjected the survival information (iie., survival time and survival status) of their corresponding clinical samples to further screen the lncRNAs associated with prognosis. The above screened mRNAs and lncRNAs related to immunity and prognosis were used for subsequent lasso model construction.

### Immune-related prognostic risk model construction

We integrated the mRNAs dataset and the lncRNAS dataset related to immunity and prognosis as well as the corresponding clinical information. We then randomly divided the integrated data set into the training set and the test set at a rate of 5:5. We used the training set to construct the lasso model, in which the Cox risk ratio model was selected to calculate the risk factor. The Cox risk proportional model was defined as:

3 $$\begin{aligned} h(t)=h_{0}(t) \times \exp \left( c_{1} x_{1}+c_{2} x_{2}+\ldots +c_{p} x_{p}\right) , \end{aligned}$$

where *t* was the survival time, $x_1$, $x_2$ to $x_p$ refers to a number of variables with predictive effect. $c_{1}$, $c_{2}$ to $c_{p}$ were the corresponding effect quantity of each variable. *h*(*t*) was a function to calculate the risk value at different times, and $h_{0}(t)$ was a function of to generate the benchmark risk. we also obtained the model error point through cross-validation. The correlation coefficients of the involved mRNAs and lncRNAs were further calculated. The formula of the lasso model was to multiply the mRNA and lncRNA involved in the construction of the model by their correlation coefficients.

According to the lasso model, we calculated the risk score of each sample. The median value of the risk score was obtained by sorting the samples. Base on the median value of scores, patients were divided into a high-risk group (risk score $\ge$ median value) and a low-risk group ( risk score < median value). We calculated the differential expression of mRNAs and lncRNAS in the distinct risk group involved in the construction of the lasso model.

We used the receiver operating characteristic curve (ROC) function to predict the survival rate based on multiple clinical features. We then drew a ROC figure over a one-year period, and calculated the area under the curve (AUC). The differences in survival status and survival time of patients in the distinct risk groups were analyzed. Finally, we analyzed the patients’ features (i.e., age, gender, and cancer stage) and the corresponding clinical information (i.e., survival time and survival status) with high-risk scores or low risk scores. By analyzing through the coxph function, we obtained the univariate and multivariate independent prognosis.

### Model-based prognosis-related immune cell analysis

We used the transcriptome data from the immune infiltration file to obtain the infiltration volume of 22 immune cells in LUAD. The difference in immune cell content between the distinct risk group was also analyzed. In addition, the immune cells with significant differences in expression between the two groups were determined (*P* < 0.05). We also calculated the median value of the expression of each immune cell in different samples. The samples were divided into a low expression group ,and a high expression group. We calculated the survival difference between the two groups to analyze the impact of immune cell expression on the patient, and discuss Survival rate and whether there was a difference (*P* < 0.05).

### Model-based differential expression analysis of genes in distinct risk groups

We divided the raw transcriptome data into distinct risk group. We calculated the differential expression of genes between these groups was according to the screening criteria of $\log (fold change) > 1$ and $P < 0.05$. We further carried out gene ontology enrichment analysis and KEGG signal pathway enrichment analysis on the selected DEGs.

### Drug screening based on differential gene expression profiles between distinct risk groups

We transformed the probe file for the differentially up and down-regulated genes of the distinct risk groups obtained by the above-mentioned difference analysis. Then, we uploaded the differential gene information to the CMap database^20^ for screening potential therapeutic compounds. According to the screening results, compounds with the values of *P* < 0.05 and enrichment score < 0 were considered as potential therapeutic compounds that reversed the LUAD gene expression profile. The compound with the highest combination score was selected for subsequent cell experiment verification. The chemical structure information of the compound was obtained in the Pubchem database^35^.

### Experimental validation of compound cytostatic properties

A549 cells were cultured in DMEM (4.5 g/L D-glucose, Gibco, USA) containing 10% fetal bovine serum, and 5,000 cells per well were seeded in a 96-well plate. After 24 hours, all the cells were stuck to the wall. We further prepared a solution of resveratrol (Lot#:C12594500, Macklin, China), methotrexate (Lot#: C12150088, Macklin, China), phenoxybenzamine (Lot#: C11947938, Macklin, China), and thiostrepton. The preparation concentration was set as 1125 mg/mg mL in dimethyl sulfoxide (DMSO, Lot#: RNBH9957, Sigma, America), and then passed through a 0.22 μm filter membrane (Merck, America). The four drugs were added at concentrations of 0, 8, 32, 64, and 128 μmol/L for 48 hours and 72 hours, respectively. After treatmented, we discarded the supernatant, added 10% CCK-8 (Lot.SX749, Dojindo, Japan) and incubated for 2 hours, and then read the results with Multiskan GO 1510-03667C at a wavelength of 450 nm.

### Statistical analysis

R Studio/R 4.1.0 is used for statistical analysis,Pearson correlation coefficient (PCC) is used to calculate the correlation, and the significant condition is set as $PCC > 0.4$ and $P < 0.001$. The univariate Cox parameter range of prognostic mRNA is $P < 0.01$. The univariate Cox of the prognostic lncRNA is *P* < 0.05. Univariate and multivariate Cox are used to analyze the relationship between clinical information and prognosis, and the test level is $\alpha = 0.05$.

## Data Availability

The datasets of this article were generated from the TCGA database.

## References

[1] Siegel RL, Miller KD, Jemal A (2019). Cancer statistics, 2019. Ca Cancer J. Clin..

[2] Gridelli C (2015). Non-small-cell lung cancer. Nat. Rev. Dis. Primers.

[3] Meza R, Meernik C, Jeon J, Cote ML (2015). Lung cancer incidence trends by gender, race and histology in the united states, 1973–2010. PloS One.

[4] Bray F (2020). Global cancer statistics 2018: Globocan estimates of incidence and mortality worldwide for 36 cancers in 185 countries (vol 68, pg 394, 2018). Ca Cancer J. Clin..

[5] Saito M, Suzuki H, Kono K, Takenoshita S, Kohno T (2018). Treatment of lung adenocarcinoma by molecular-targeted therapy and immunotherapy. Surg. Today.

[6] Stella GM, Luisetti M, Pozzi E, Comoglio PM (2013). Oncogenes in non-small-cell lung cancer: Emerging connections and novel therapeutic dynamics. Lancet Respir. Med..

[7] Sun G, Zhao T (2020). Lung adenocarcinoma pathology stages related gene identification. Math. Biosci. Eng..

[8] Bhan A, Soleimani M, Mandal SS (2017). Long noncoding rna and cancer: A new paradigm. Cancer Res..

[9] Zhou J (2018). Long noncoding rna casc9. 5 promotes the proliferation and metastasis of lung adenocarcinoma. Sci. Rep..

[10] Ruiz-Cordero R, Devine WP (2020). Targeted therapy and checkpoint immunotherapy in lung cancer. Surg. Pathol. Clin..

[11] Duan J (2020). Refined stratification based on baseline concomitant mutations and longitudinal circulating tumor dna monitoring in advanced egfr-mutant lung adenocarcinoma under gefitinib treatment. J. Thorac. Oncol..

[12] Xu J-Y (2020). Integrative proteomic characterization of human lung adenocarcinoma. Cell.

[13] Li Y (2017). Prognostic alternative mrna splicing signature in non-small cell lung cancer. Cancer Lett..

[14] Kaishang Z (2018). Elevated expression of twinfilin-1 is correlated with inferior prognosis of lung adenocarcinoma. Life Sci..

[15] Chiou J (2017). Decrease of fstl1-bmp4-smad signaling predicts poor prognosis in lung adenocarcinoma but not in squamous cell carcinoma. Sci. Rep..

[16] Liu C (2019). Identification of a novel glycolysis-related gene signature that can predict the survival of patients with lung adenocarcinoma. Cell Cycle.

[17] Jiang H, Xu S, Chen C (2020). A ten-gene signature-based risk assessment model predicts the prognosis of lung adenocarcinoma. BMC Cancer.

[18] TCGA. The cancer genome atlas (tcga) database. Website (2021). https://www.cancer.gov/tcga.

[19] Zhang, K. & Hong, W. Cancer genome atlas pan-cancer analysis project. *Zhongguo Fei Ai Za Zhi* **18** (2015).

[20] Lamb J (2006). The connectivity map: Using gene-expression signatures to connect small molecules, genes, and disease. Science.

[21] Lin K (2020). A comprehensive evaluation of connectivity methods for 1000 data. Brief Bioinform..

[22] Zhang L (2021). Connectivity mapping identifies bi-2536 as a potential drug to treat diabetic kidney disease. Diabetes.

[23] Bhattacharya S (2018). Immport, toward repurposing of open access immunological assay data for translational and clinical research. Sci. Data.

[24] Li, Z., Mao, K., Ding, B. & Xue, Q. Integrative analysis of multi-omics data identified klrc3 as key nodes in a gene regulatory network related to immune phenotypes in lung adenocarcinoma. Preprints.

[25] Cho H (2014). Mica/b and ulbp1 nkg2d ligands are independent predictors of good prognosis in cervical cancer. BMC Cancer.

[26] Geng W (2021). Identification of the prognostic significance of somatic mutation-derived lncrna signatures of genomic instability in lung adenocarcinoma. Front. Cell Dev. Biol..

[27] Wang L (2019). Systematic identification of lincrna-based prognostic biomarkers by integrating lincrna expression and copy number variation in lung adenocarcinoma. Int. J. Cancer.

[28] Ma J (2010). The m1 form of tumor-associated macrophages in non-small cell lung cancer is positively associated with survival time. BMC Cancer.

[29] Ljunggren H-G, Malmberg K-J (2007). Prospects for the use of nk cells in immunotherapy of human cancer. Nat. Rev. Immunol..

[30] Malaguarnera L (2019). Influence of resveratrol on the immune response. Nutrients.

[31] Fan Y (2020). Resveratrol modulates the apoptosis and autophagic death of human lung adenocarcinoma a549 cells via a p53-dependent pathway: Integrated bioinformatics analysis and experimental validation. Int. J. Oncol..

[32] Guo M (2020). Autologous tumor cell–derived microparticle-based targeted chemotherapy in lung cancer patients with malignant pleural effusion. Sci. Transl. Med..

[33] Yoham, A. L. & Casadesus, D. Phenoxybenzamine. StatPearls [Internet] (2021).

[34] Inchiosa MA (2018). Anti-tumor activity of phenoxybenzamine and its inhibition of histone deacetylases. PloS One.

[35] Pubchem. The pubchem database. Website (2021). https://pubchem.ncbi.nlm.nih.gov.

